# Flexible Robust and High‐Density FeRAM from Array of Organic Ferroelectric Nano‐Lamellae by Self‐Assembly

**DOI:** 10.1002/advs.201801931

**Published:** 2019-01-28

**Authors:** Mengfan Guo, Jianyong Jiang, Jianfeng Qian, Chen Liu, Jing Ma, Ce‐Wen Nan, Yang Shen

**Affiliations:** ^1^ School of Materials Science and Engineering State Key Lab of New Ceramics and Fine Processing Tsinghua University Beijing 100084 China; ^2^ Center for Flexible Electronics Technology Tsinghua University Beijing 100084 China

**Keywords:** ferroelectric random access memories (FeRAMs), grain boundaries, P(VDF‐TrFE), self‐assembly, thermal stability

## Abstract

Ferroelectric memories are endowed with high data storage density by nanostructure designing, while the robustness is also impaired. For organic ferroelectrics favored by flexible memories, low Curie transition temperature limits their thermal stability. Herein, a ferroelectric random access memory (FeRAM) is demonstrated based on an array of P(VDF‐TrFE) lamellae by self‐assembly. Written data shows enhanced thermal endurance up to 90 °C and undergoes 12 thermal cycles between 30 and 80 °C with little volatilization. The promoted thermal stability is attributed to pinning effect at interfaces between grain boundaries and lamellae, where charged domain walls and charged defects are coupled. These results provide a strategy for improving robustness of organic flexible FeRAMs, and reveal an attracting coupling effect between different phases of ferroelectric polymer.

Ferroelectric random access memory (FeRAM) is a novel class of nonvolatile memory storage device with low‐power consumption.^1^ A thin layer of ferroelectric materials is introduced in FeRAM, functioning in write‐in, read‐out, and nonvolatile storage. Therefore, binary information is encoded by external electric field, as ferroelectric polarization can be tuned into up and down orientation, representing “1” and “0” states. Moreover, the polarization can be retained after removal of external field, giving rise to the nonvolatility of FeRAM.^2^, ^3^ Typically, inorganic perovskites have been selected as the ferroelectric layer owing to their high relative permittivity,^4^ stable polarization,^5^ and ability to be constructed into thin film with a few unit cells.^6^ However, while flexible materials are favored due to their potential application in wearable devices and opportunity for novel physical phenomena, the compatibility of inorganic materials with flexible FeRAM devices is limited by the intrinsic brittleness and high processing temperature.^7^, ^8^ Therefore, organic ferroelectrics stand out for their easy processability, low‐cost, and especially the low processing temperature.

Poly(vinylidene fluoride) (PVDF) and its copolymers are one of the most attractive families of organic ferroelectric materials, and have been utilized for nonvolatile memories,^9^ ferroelectric field‐effect transistors,^10^ energy storage capacitors,^11^ and wearable electrocaloric cooling‐system.^12^ There are four polymorphs of PVDF‐based polymers with different chain conformations, e.g., the paraelectric α‐phase (TG^+^TG^−^) and the ferroelectric β‐phase (all‐trans), γ‐phase (TTTG^+^TTTG^−^), and δ‐phase (TG^+^TG^−^), where T (trans) and G^±^ (gauche^±^) refer to the torsional bond arrangements with substituents at 180° and ±60° to each other, respectively. Among the ferroelectric phases, β‐phase PVDF exhibits the strongest ferroelectricity because the dipolar moments induced by the alternating —CF_2_ and —CH_2_ groups are strictly perpendicular to the polymer chains in all‐trans conformation.^13^ Incorporation of trifluoroethylene (TrFE) groups in the PVDF chains promotes the formation of the all‐trans conformation, therefore the corresponding P(VDF‐TrFE) copolymer features higher content of β‐phase during annealing and the strongest ferroelectricity among all PVDF‐based polymers.^14^, ^15^, ^16^ Besides, P(VDF‐TrFE) usually crystallizes into lamellae with large aspect ratio,^17^ where polymer chains are aligned parallel to lamellae normal. Considering that the primary dipolar moments are perpendicular to the axial direction of the chains, better ferroelectric performances will be obtained when lamellae are edge‐on in P(VDF‐TrFE),^9^ which is highly desirable for FeRAM applications.

As a result, tremendous efforts have been put forward to fabricate oriented P(VDF‐TrFE) for nonvolatile memory storage. For instances, Park et al. have managed to induce highly oriented P(VDF‐TrFE) by epitaxial growth of lamellae on different substrates, including textured poly(tetrafluoroethylene) (PTFE),^18^ graphene electrode,^10^ and semiconducting rubrene single crystal.^19^ The advantage of epitaxial growth is that the strong interactions between the substrates and P(VDF‐TrFE) lead to highly ordered structure. Recently, Zhu and co‐workers employed removable PTFE substrate to induce the self‐assembly of P(VDF‐TrFE) into array of edge‐on lamellae.^20^ The corresponding P(VDF‐TrFE) films of ordered structure exhibit much enhanced ferroelectric performances compared with their counterparts of random orientation of lamellae.

Mechanical stress is another path to fabricate oriented P(VDF‐TrFE) for flexible FeRAM. Park and co‐workers demonstrated P(VDF‐TrFE) memory from effective alignment by shear stress,^21^ as applied stress reorients and reorganizes polymer chains.^22^, ^23^ Hu et al. have proposed nanoimprint lithography (NIL), which offers stress‐assisted alignment and patterns of polymers.^24^ Alignment is facilitated by pressing polymers into molds with nanoconfinements. During crystallization of P(VDF‐TrFE) within these nanoconfinements, the growth of lamellae induces mechanical stress which in return orients the P(VDF‐TrFE) chains,^25^, ^26^ and as‐fabricated P(VDF‐TrFE) films exhibit low operation voltage and rapid switching.^9^ Moreover, NIL achieved high data density films by realizing confinements down to nanometers. Molds with regular nanostructure have been prepared by electron beam lithography and reactive ion etching in the work of Hu et al.^9^ Shen and co‐workers further enhanced the data density by imprinting anodized aluminum foils.^27^ The above methods applied in advanced nonvolatile memories have confirmed the outstanding performance of P(VDF‐TrFE) as a candidate for flexible FeRAM with good performances.

Beneficial from the low‐melting temperature, processing temperature of the P(VDF‐TrFE) during epitaxial growth, recrystallization, and mechanical stressing is notably lower than that of inorganic ferroelectrics. However, as a result of relatively low Curie temperature around 110 °C,^14^ the stability issues of ferroelectric polarization in PVDF‐based FeRAM at elevated temperatures have to be addressed. As the ferroelectricity within nanoscale features will be further weakened and methods to examine thermal stability become more difficult down to nanometers, the thermal stability issues of high‐density organic FeRAM become more prominent.^28^ Investigation of new systems, e.g., molecular ferroelectrics,^29^, ^30^ is promising to overcome the low temperature Curie transition. Xiong et al. have successfully synthesized molecular ferroelectric crystal with Curie temperature of 153 °C.^31^ Lately, single phase organic–inorganic perovskite ferroelectric with Curie temperature of 133 °C^32^ and a family of metal‐free 3D perovskite ferroelectrics with highest Curie temperature of 175 °C^33^ have been fabricated by the same group, which reveal the possibility to overcome the low Curie temperature in organic ferroelectrics. Still, for PVDF‐based ferroelectric polymers, there are inspiring works showing us some new paths to solving the thermal stability issues. Zhu and co‐workers have conducted atomic force microscope (AFM) tip deflection‐temperature experiments and verified that P(VDF‐TrFE) epitaxially grown on PTFE is more thermally stable than P(VDF‐TrFE) on a silicon wafer.^34^ Jonas and co‐workers also proved that the thermal stability of a mechanically oriented P(VDF‐TrFE) through NIL is not suppressed compared to bulk P(VDF‐TrFE), by resolving the piezoresponce force microscope (PFM) phase images at different sample temperature.^28^ Shen and co‐workers have achieved better data stability in P(VDF‐TrFE) with modified VDF/TrFE ratio, which introduces defects to control polarization reversals.^35^ To conclude, these works demonstrate strong correlation between better thermal stability and aligned, defect‐mediated P(VDF‐TrFE). Although FeRAMs with enhanced thermal stability are presented, the detailed mechanism is missing.

Herein, we demonstrate that a FeRAM with high thermal stability and data storage density of ≈60 GB inch^−2^ could be achieved from an array of edge‐on nano‐lamellae by low‐temperature self‐assembly of P(VDF‐TrFE). Instead of mechanical stress, texture is induced by the self‐assembly of nano‐lamellae during crystallization with the aid of a removable PTFE substrate. Compared to a spin‐coated FeRAM device with thermal endurance of 70 °C, the self‐assembled FeRAM exhibits maximum thermal stability up to at least 90 °C. Minor volatilization is observed for the FeRAM even after 12 thermal cycles between 30 and 80 °C. The enhanced thermal stability is attributed to its periodical package of crystalline and grain boundaries in the texture, where charged domain walls and charged defects are observed at grain boundaries. The coupling effect between them results in the enhanced thermal stability of robust self‐assembled FeRAM.

The process and mechanism of self‐assembly is illustrated in **Figure** **1**a. For the solution‐processed P(VDF‐TrFE) films, the lamellae are randomly oriented in thick films (Figure S1a, Supporting Information) and tend to stack layer‐by‐layer with their normal axis aligned parallel to films normal as the thickness of the films decreases (Figure 1b). To induce the edge‐on conformation of P(VDF‐TrFE) lamellae, a removable PTFE substrate is attached on to the surface of the solution‐processed P(VDF‐TrFE) films. The PTFE/P(VDF‐TrFE) bilayer is then subjected to hot‐pressing at 150 °C 0.5 MPa. During the recrystallization process of P(VDF‐TrFE), two kinds of molecular interactions between the PTFE and P(VDF‐TrFE) induce self‐assembly of P(VDF‐TrFE) chains into array of edge‐on lamellae (as illustrated in Figure 1c). The first one exists at the interface of PTFE and P(VDF‐TrFE),^18^, ^36^ denoted by the blue rectangle in Figure 1c. The surface of PTFE film is fluorinated, as the main chain of PTFE is fully bonded to fluorine atoms. This fluorinated surface could attract hydrogen atoms and repel fluorine atoms of P(VDF‐TrFE) by electrostatic force. Consequently, at the interface, molecules of P(VDF‐TrFE) will be guided parallel to the film surface by PTFE. The second interaction is within P(VDF‐TrFE), which actuates the intensive crystallization and endow crystallized P(VDF‐TrFE) with fine ferroelectricity,^14^ denoted by the red rectangle in Figure 1c. Combining the two kinds of interactions, we propose that ordered structure is induced, as P(VDF‐TrFE) chains near the interface are aligned along the in‐plane directions of the film and then P(VDF‐TrFE) crystallize from the interface to another face. Therefore, the formation of ordered structure is mainly driven by intermolecular interaction, which is essential for the self‐assembly process.^37^ It is also worth noting that the intermolecular interactions are effective within certain distances from the P(VDF‐TrFE)/PTFE interface. In thin films, the edge‐on lamellae span throughout the thickness direction (Figure 1d,e; Figure S2d, Supporting Information). For thick films, the self‐assembly is applied to one side or both sides, and the result showed that the vertical lamellae could be induced to a maximal ≈6 µm from the P(VDF‐TrFE)/PTFE interface (Figure S1, Supporting Information). For simplification, samples without self‐assembly process are denoted as not self‐assembled (NSA) ones, and self‐assembled samples are denoted as self‐assembled (SA) ones.

**Figure 1:**
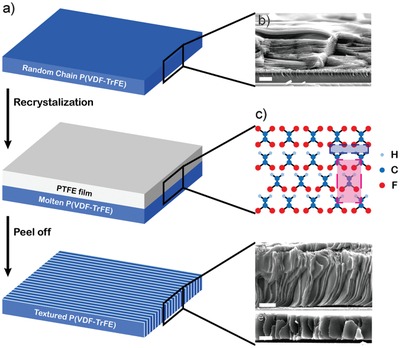
Fabrication of self‐assembled P(VDF‐TrFE) with induced array of lamellae. a) Schematic diagram of self‐assembly method. b) Cross‐sectional SEM images of films before self‐assembly. c) Possible mechanism of self‐assembly method inducing ordered structure. The blue rectangle indicates interaction of PTFE and P(VDF‐TrFE) at the interface, and the red rectangle indicates the interaction between P(VDF‐TrFE) chains. d,e) Cross‐sectional SEM images of films after self‐assembly. Scale bars: 1.5 µm.

Within a single lamella, the P(VDF‐TrFE) chains are aligned along out‐of‐plane direction of the lamella, folding back and forth.^14^ Irregular components including loose loops, tie segments, and dangling ends are mostly located between neighboring lamellae,^23^, ^38^ namely the grain boundaries. Therefore, from the lamellar structure in SA films, we can conclude that the chain direction of polymer is aligned parallel to the film surface by the self‐assembly. Considering that the dipole in P(VDF‐TrFE) rotates about the *c*‐axis, or chain axis, on applying electric field, the films with edge‐on lamellae exhibit more effective switching of polarization.

After the recrystallization processing during annealing, the dominant phase is ferroelectric β‐phase in both SA and NSA films. The diffraction peaks at 19.8° corresponding to the (110)/(200) diffraction in β‐phase^39^ are observed in X‐ray diffraction (XRD) profiles (Figure S3a, Supporting Information). Pseudohexagonal patterns are also obtained by selected area electron diffraction (SAED) (Figure S3b, Supporting Information), corresponding to the β‐phase.^14^, ^17^ To verify the ordered structure in SA films more accurately, 2D wide angle X‐ray diffraction (2D‐WAXD) experiments have been conducted and the experimental configuration is illustrated in Figure S3c (Supporting Information). NSA films with random chain alignment exhibit a ring with uniform intensity in their 2D‐WAXD patterns (shown in Figure S3d,e, Supporting Information), corresponding to the (110)/(200) diffraction. By contrast, a split ring with six well‐oriented arc was observed in SA P(VDF‐TrFE), as shown in **Figure** **2**a, indicating preferential orientations of lamellae. By integrating the intensity along rotational angle, 1D‐WAXD profile is obtained. As shown in Figure S3h (Supporting Information), the two stronger peaks and the four weaker ones are attributed to (200) and (110) diffractions, respectively. The stronger peaks are at horizontal position, implying that (200) lattice planes are mainly aligned normal to horizontal direction, and parallel to the film surface. The diffraction peak of P(VDF‐TrFE) in 2D‐WAXD experiment is calculated to be 19.8° by integrating intensity along diffraction angle (Figure S3i, Supporting Information), same as the XRD profile. Moreover, the Fourier transform IR (FTIR) spectrum also reveals lamellar alignment in SA films. As the characteristic peak of β‐phase P(VDF‐TrFE) at 1288 cm^−1^ (symmetric stretching vibration of CF_2_) is observed in both SA and NSA films,^39^ the 1400 cm^−1^ band corresponds to CH_2_ wagging vibration with dipole moment along *c* axis,^27^ the weaker intensity in SA film indicates that polymer chains in lamellae are aligned parallel to the film surface (see Figure S4a, Supporting Information).

**Figure 2:**
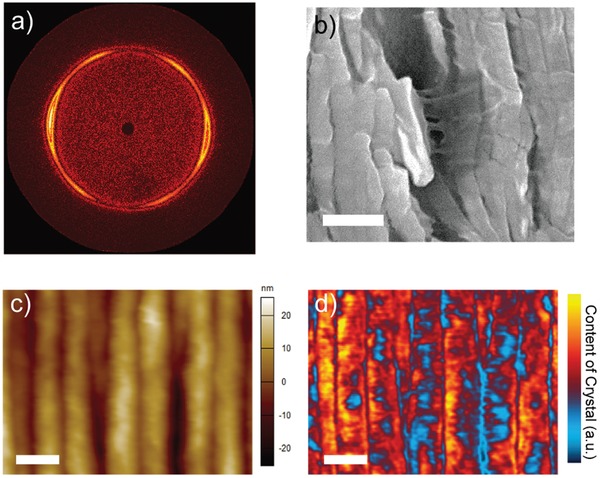
Structural characterization of P(VDF‐TrFE) films. a) 2D‐WAXD identification of a SA film. The X‐ray beam was directed parallel to the film surface. b) Detailed cross‐sectional SEM image of cracked lamellae in a SA film, with ductile grain boundary connecting brittle lamellae. c) AFM topography and d) AFM‐IR absorbance image of a SA film. Less absorbance between neighboring lamellae indicates less crystal component at grain boundaries. Scale bars: 200 nm.

It is interesting that ductile polymer matter connecting cracked brittle lamellae is observed in SA films, after electron irradiation during scanning electron microscope (SEM) characterization (Figure 2b). We note that the ductile component is grain boundary with random chain conformation and disordered chain package, adjacent to neighboring lamellae. Hence, we have conducted an in situ AFM‐based infrared spectroscopy (AFM‐IR) study of SA films (Figure 2c,d). In Figure 2c, the ordered edge‐on lamellae are observed in the AFM topography image. AFM‐IR spectra are then collected by stimulating a laser beam onto the same area and probing points of interests with varying wavenumbers. In the AFM‐IR spectrum (shown in Figure S4b, Supporting Information), the characteristic peak of β‐phase (PVDF‐TrFE) centered at 1288 cm^−1^ is detected, in good agreement with the FTIR spectrum (Figure S4a, Supporting Information). The peak intensity is related to the content of β‐phase in the area of interest probed. The lower intensity of the peak at 1288 cm^−1^ indicates that smaller amount of β‐phase P(VDF‐TrFE) is present in the grain boundaries between the edge‐on lamellae compared to the center of the lamella, as shown in Figure S4b (Supporting Information). Distribution of β‐phase P(VDF‐TrFE) among grains and grain boundaries in the SA films are then visualized by mapping of the intensity of the peak corresponding to the C—F bond deformation in β‐phase in the AFM‐IR spectra at 1288 cm^−1^ (Figure 2d). The blue color in the trenches between two neighboring lamellae indicates that less β‐phase are concentrated within these areas. This evidence supports the assumption that the polymer chains mostly take the random conformation inside the grain boundaries. The results of AFM‐IR also agree with the previous observations that the amorphous phase consisting of dangling chains, TG^+^, or TG^−^conformation and loose loops exists in crystallized P(VDF‐TrFE).^23^ It has been reported that the ferroelectric behavior of amorphous component is different from its crystal counterpart.^23^, ^40^ In SA P(VDF‐TrFE), the amorphous and crystal phases are finely separated, providing a delicate platform to investigate their ferroelectricity.

Voltage pulses on PFM tip have generated ferroelectric switching in a SA film, and then the reversal domains are detected by PFM. **Figure** **3**b shows a series of typical reversal domains on surface of well‐oriented lamellae in Figure 3a. The five switched domains are generated by −10 V pulse with 5, 50, 100, 200, and 500 ms, respectively. Generally, shapes of switched domains are rectangles with their width and length perpendicular and parallel to grain boundary. In addition, borders of reversal domains along grain boundary are smooth. These results are different from pulse induced domains on homogeneous films, where shapes of switched domains are circular with rough borders.^41^, ^42^, ^43^

**Figure 3:**
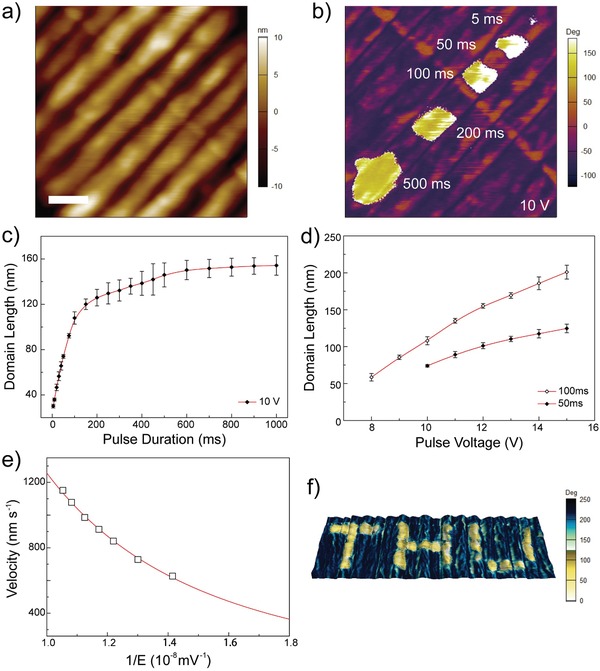
Stimuli of reversal domains in SA ferroelectric polymer as an application of FeRAM. a) AFM topology and b) PFM phase images of SA P(VDF‐TrFE). The five reversal domains in (b) are stimulated with voltage pulses of same amplitude at 10 V and increasing duration at 5, 50, 100, 200, 500 ms, respectively. The scale bar is 150 nm. Numeric figures of domain lengths as a function of c) pulse duration and d) amplitude in SA films, and e) the dependence of domain lengths on electric field. The pulse amplitude was fixed at 10 V in (c) and pulse duration was fixed at 50 and 100 ms in (d). f) Illustration of FeRAM application in SA P(VDF‐TrFE). The uniform background was polarized with a positive d.c. voltage, and the letters were stimulated with several negative voltage pulses (−10 V, 100 ms).

The rectangular shape and smooth borders can be explained by pinning effect at grain boundary, which impedes domain wall motions.^44^, ^45^ Pinning potential is enhanced and hence hinders domain wall motion at grain boundaries, as the ferroelectricity of grain boundaries is different from that of lamellar internal, because of the higher density of structural and electric defects induced by loose loops, tie segments, as well as dangling ends. With prolonged voltage pulse, domain walls propagated and contacted grain boundary. Then, pinning effect confined propagation in direction vertical to grain boundary, resulting in smooth borders at interface of lamellae and amorphous boundaries. Only when pulse voltage was higher than the threshold or prolonged duration reached a critical value, could the domain wall go through a pinning–depinning transition to continue propagating, as revealed by the reversal domain stimulated by pulse −10 V, 500 ms in Figure 3b, whose domain wall slightly crosses the grain boundary and extends into the neighboring grain.

The kinetics of domain wall motion is then investigated. The distribution of electric field (*E*) around a sphere tip of radius (*R*) is modeled as^23^

(1) $$E\left(r\right)\;=\frac{VR}{d\sqrt{r^{2}+R^{2}}}$$

where *V* is tip bias and *d* is the film thickness. The propagating velocity of domain wall (*v*) is approximated as

(2) $$v\;\left(r\right)=\frac{r_{n+1}-r_{n}}{t_{n+1}-t_{n}}$$

where *n* is a serial number and *r*  = (*r_n_* + *r*_*n* + 1_) /2. Thus, we can obtain the dependence of the domain wall velocity on electric field (Figure 3d), which fit well to the creep model given as^45^

(3) $$v\;=v_{\infty }\;\exp\left[-{\left(\frac{E_{0}}{E}\right)}^{\mu }\right]$$

where *E*_0_, *v*_∞_, μ are the depinning electric field, upper limit of domain wall propagating velocity, and a dynamical exponent, respectively. The dependence of average domain length on pulse duration and voltage are presented in Figure 3c,d. As seen, the average domain length increases abruptly with prolonged pulse width and steadily with increasing pulse voltage. The fitted results are presented in Figure 3e. The exponent *µ* is determined to be 0.27 in the SA films. The value is consistent with previously reported results in defect‐free P(VDF‐TrFE) nanomesa and LB films,^41^, ^42^ and close to the predicted value of 0.25 for 1D domain wall.^45^ 2D domain wall with *µ* = 0.5 has been reported within an aligned nanostripes,^23^ where two‐dimensionality is attributed to strong pinning effect caused by interfaces between amorphous component and lamellae. Compared with the aligned vertical grain boundaries in nanostripes, the short‐ranged pinning potential of the interfaces has been eliminated within single lamella. Instead, long‐ranged elastic energy dominates the propagation owing to the collective switching of polymer chains. The combination of long range elastic energy and low pinning potential gives rise to the 1D domain wall in SA P(VDF‐TrFE).

Given the well size‐dependence of square domains on the amplitude and duration of voltage pulses, a prototype FeRAM is demonstrated in the SA P(VDF‐TrFE) films. As shown in Figure 3f, data are stored in “bit” of 100 × 100 nm^2^ by voltage pulses of −10 V at 100 ms. With each bit of 100 × 100 nm^2^, memory density of this SA FeRAM is at least 60 GB inch^−2^. In addition to the high memory density, robust memory retention and fatigue resistance performances are also observed for the SA FeRAMs (Figure S5, Supporting Information). The written patterns remain nearly the same after a month at room temperature in ambient atmosphere, with domain borders changing from vague to clear which might be caused by relaxation of ferroelectric polymer chains. Moreover, domain walls overlapping with grain boundaries are still smooth in mosaic pattern after a month of retention, due to the motion hindrance of the pinning effects (see Figure S5a,b, Supporting Information). Cycling of ferroelectric polarization switching is performed up to 100 times with switching spectrum (SS) PFM. 180° switching of ferroelectric polarization in the square domain is still observed after the cycling tests, indicating robust fatigue‐resistance of the SA FeRAM (Figure S5c, Supporting Information).

For thermal stability test, both the SA and NSA films are attached to a heating stage with identical domain patterns written onto their surface at 25 °C (**Figure** **4**a; Figure S6b, Supporting Information). Then the temperature is ramped up to 100 °C. The remaining area of the reversal domains is recorded and measured every 10 °C during ramping process. In principle, the SA films exhibit better thermal stability of ferroelectric domains compared to the NSA films. As shown in Figure 4c, with increasing temperature, the domain patterns of the SA films remain fairly stable up to ≈80 °C, beyond which rapid depolarization of the ferroelectric domains is observed as indicated by fading of the patterns. The domain patterns are completely thermal‐bleached when the temperature increases to 100 °C. As for the NSA films, thermally activated depolarization process is observed immediately from room temperature as suggested by the monotonic decrease in residual area ratio with increasing temperature. Beyond a lower critical temperature of ≈70 °C, the domain patterns dramatically fade and completely disappear at 90 °C compared to 100 °C for their SA counterparts. Thermal cycling tests are also performed on both SA and NSA films between 30 and 80 °C. The remaining area of the six square domains (as shown in Figure S7b,f, Supporting Information) is measured after each cycle and plotted as function of cycle times in Figure 4d. Two striking features could be readily observed from the results of thermal cycling tests. First, the domain patterns and residual area ratio of the SA films remain almost unchanged after at least 12 cycles (see also Figure S7c,d, Supporting Information). Second, for the NSA films, the residual area ratio decreases rapidly to ≈25% after the first 3 cycles and remains stable during the rest of the thermal cycles. It is worth noting that, for the SA films, the domain walls overlapping with grain boundaries are still tightly pinned even at 90 °C (see Figure 4b). Thermally activated depolarization occurs preferentially in the direction parallel to grain boundary. These features suggest that the superior thermal stability of the SA films might be associated to the strong pinning effect at the grain boundary.

**Figure 4:**
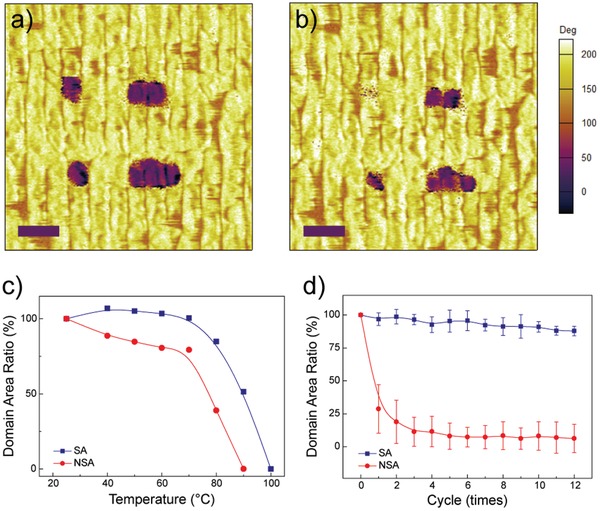
Enhanced thermal stability in SA P(VDF‐TrFE). a–c) PFM images of data stored in self‐assembled film at a) 25 °C and b) 90 °C, as well as c) numeric figure of residual area of reversal domains as a function of elevated temperature in a SA film (blue) and a NSA film (red). d) Numeric figure of residual area of reversal domains as a function of thermal cycles in a SA film (blue) and a NSA film (red). Scale bars: 200 nm.

We are motivated to investigate the origin of pinning effects in grain boundaries of the SA films. By scanning the domain patterns in in‐plane PFM (IP‐PFM) mode, we are able to observe in‐plane stripe domains at grain boundaries (**Figure** **5**a,b). In‐plane phase image (Figure S8b,d, Supporting Information) further reveals that these domains are 180°, indicating that these in‐plane domain walls are charged by polarons,^46^ i.e., C—H and C—F dipoles, at the interfaces between amorphous grain boundaries and crystal lamellae. Space‐resolved ferroelectric switching of the dipoles within the edge‐on lamellae is then investigated by SS‐PFM. Switching spectra from 9 points spanning across two neighboring edge‐on lamellae (see Figure S9a, Supporting Information) are collected. For all the switching spectra, additional peaks can be observed at voltage stimuli around ±35 V (highlighted in yellow in Figure S9b, Supporting Information), lower than the voltage required for the switching of ferroelectric dipoles in the edge‐on lamellae (as shown by the two peaks highlighted in blue and red). The spectra are then fitted and additional peaks are extracted by subtraction of spectra with common butterfly shapes. The extracted peak amplitude and calculated imprint from fitted switching spectra at different points are presented in Figure 5c. As the PFM tip moves from grain boundaries (red area in Figure 5c) to lamellae centers (blue area in Figure 5c), amplitude of additional peaks fluctuates around 120 pm (see red line). The low‐voltage‐stimulated peaks could be related to the ferroelectric response of amorphous grain boundaries, which can be covered by PFM tip and involved in ferroelectric switching. As ferroelectric polarons are more disordered and of smaller amount in amorphous grain boundaries, ferroelectric response of grain boundaries appears at lower voltage compared to its crystal counterparts. Moreover, after ferroelectric response of amorphous components at ≈35 V, the tip voltage increases to 90 V, during which charge/hole injection will happen and injected charge/hole will be left after removal of tip voltage.^47^

**Figure 5:**
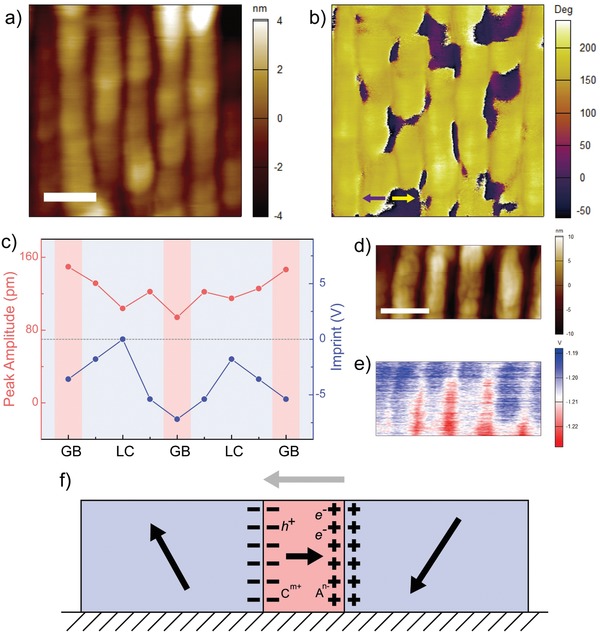
Mechanism of improved stability in SA P(VDF‐TrFE). a) AFM topography, b) PFM in‐plane phase images of SA P(VDF‐TrFE) after retention. Lateral polarization in yellow area points to the left, and lateral polarization in purple area points to the right. Stripe‐shaped antidomains can be observed at grain boundaries, indicating discontinuous polarization and charged domain walls. The scale bar is 150 nm. c) Extracted imprint and extra peak amplitude from SS‐PFM spectra collected from points at grain boundaries (GB) to lamellar centers (LC). d) AFM topography and e) SKPM surface potential images of a unpoled SA film. Surface potential at grain boundaries is lower. The scale bar is 100 nm. f) Schematic illustration of coupling between charged domain wall and charged defects at grain boundary. The black arrows denote the possible polarization in lamellae and grain boundary, and the grey arrow denotes the depolarization field brought by the charged domain walls.

In addition, ferroelectric imprint deviates from 0 V when the AFM tip moves close to grain boundaries (a maximal deviation of −7.2 V) and returns to ≈0 V at the center of lamella (see blue line). Generally, ferroelectric imprint is attributed to the screening of charged defects within switching area by bottom electrode.^48^ With more charged defects, i.e., impurity ions, vacancies, the ferroelectric imprint deviates more substantially from 0 V. In PVDF‐based polymers, impurity ions are the major defects, and are expected to be concentrated into amorphous phase where chains are loosely packed. The concentrated charged defects lead to the larger imprint observed at grain boundaries. Furthermore, scanning Kelvin probe microscope (SKPM) experiment is conducted, which can detect film surface potential by analyzing electrostatic interaction between tip on bias and area under it. By scanning the area in Figure 5d, we find out that the surface potential decreases evidently by ≈0.2 V at grain boundaries (see Figure 5e), owing to higher content of charged defects within amorphous phases. While detecting a laid down lamella, potential of amorphous lamellar surface also decreases comparing to the bottom electrode and isolated nanostripes (see Figure S10, Supporting Information), confirming the existence of concentrated impurity ions at grain boundaries.

As charged domain wall and charged defects are both detected at grain boundaries, the interplay between them cannot be neglected. As illustrated in Figure 5f, with the presence of charged domain wall, depolarization field is generated within grain boundary as indicated by the grey arrow. This built‐in electric field induces migration of the charged defects, including injected charge/hole and impurity ions, which in return screens the uncompensated charges brought by head‐to‐head or tail‐to‐tail polarization.^49^ In consequence, the ferroelectric polarization written by tip voltage is tightly pinned at the 2D interfaces between crystallized lamellae and amorphous grain boundaries, resulting in the enhanced thermal stability of SA FeRAMs.

It is worth noting that these coupling effects between charged domain walls and charged defects also explain why enhanced performance can be obtained in P(VDF‐TrFE) with aligned structure, by mechanical stress or epitaxial growth. Moreover, the coupling effects do not only exist in SA P(VDF‐TrFE), but also in annealed P(VDF‐TrFE), where pinned ferroelectric domain walls overlap with the curvy grain boundaries.^41^, ^42^, ^43^ The advantage of self‐assembly is to present an ordered structure with crystal and amorphous phases distinctly separated. The ferroelectricity of these components is different but coupled, leading to the enhanced thermal stability and robust FeRAMs.

In conclusion, we demonstrate a simple self‐assembly method to fabricate an ordered structure in P(VDF‐TrFE). The self‐assembled P(VDF‐TrFE) exhibits high storage density of 60 GB inch^−2^ as a prototype of flexible FeRAM. We experimentally determine our self‐assembled FeRAM stored data more robustly, with temperature endurance enhanced over 10 °C and reliable thermal cycling ability. Detailed SS‐PFM and SKPM profiles show that the enhanced stability of self‐assembled FeRAM is based on coupling effects between charged domain wall and charged defects at the interface of grain boundary and lamella. This work shows us a novel path to address the thermal stability issues in organic FeRAMs. And for the first time, a detailed analysis about the origin of enhanced performance in aligned P(VDF‐TrFE) is presented.

## Experimental Section

*Materials and Methods*: The P(VDF‐TrFE) 70/30 mol% copolymer was obtained from Piezotech and used as‐received. The P(VDF‐TrFE) was dissolved into methyl ethyl ketone at different concentrations ranging from 1% to 5% w/v depending on designed film thickness. P(VDF‐TrFE) films were first spin‐coated onto commercially available silicon wafers with platinum coating layer. The spin speed was set at 4000 rpm, and film thickness varied from 80 nm to 1 µm. Then the films were dried in a vacuum oven at 30 °C for 1 h. For SA films, the dried films were capped by a commercially available PTFE film and hot‐pressed at 150 °C 0.5 MPa for 30 min, prior to peeling off the PTFE film. Then the P(VDF‐TrFE) films were annealed at 150 °C to obtain better morphology. For NSA films, the dried films were directly annealed in the same condition as the SA films. Thicker films were fabricated by hot‐pressing a nonwoven fabric of P(VDF‐TrFE). P(VDF‐TrFE) solution with 10% w/v concentration was electrospun to obtain the fabrics. The thick SA films were prepared by the same method applied to SA films on silicon wafers. Thick NSA films were first hot‐pressed to PET films at 150 °C 0.5 MPa for 30 min and then annealed.

*Morphology and Structure Characterization*: The cross‐sectional morphology of the SA and NSA films with different thickness was characterized with a commercial SEM (Zeiss GeminiSEM 500). The surface morphology of the SA and NSA films was characterized in contact mode with a commercial AFM (Asylum Research MFP‐3D), using Si cantilevers with Pt/Ir coating (NanoWorld Arrow‐CONTPt). To obtain structure information, XRD was performed on a Rigaku D/max 2500 PC diffractometer with Cu Kα radiation. SAED was carried out using transmission electron microscopy (JEOL2011). WAXD experiments were performed on a Bruker D8 Discover diffractometer with GADDS area detector. The X‐ray was selected as Cu Kα radiation. To confirm the chain conformation, FTIR spectroscopy was carried out on Nicolet 6700 under reflection mode. AFM‐IR spectroscopy was performed on an Anasys NanoIR2. The tips supplied by Anasys were custom made for the NanoIR2. All AFM‐IR experiments were performed under contact mode. For single spectrum data, the resolution was set to 2 cm^−1^, and each wavenumber point was averaged over 16 pulses. For IR mapping, wavenumber of IR beam was fixed at 1288 cm^−1^. The scan rate was set at 0.1 Hz, with each point being the average from 16 pulses.

*Ferroelectric and Electric Property Characterization*: A commercial scanning probe microscope (Asylum Research MFP‐3D) was applied for PFM and SKPM measurements. In PFM testing, a Si cantilever with conducting Pt/Ir coating layer of spring constant 0.2 N m^−1^ (NanoWorld Arrow‐CONTPt) was used. For out‐of‐plane PFM imaging, tips were modulated at 60 kHz with an AC voltage of 3 V. Before the tip‐induced switching, the selected area was first poled with 12 V DC tip bias. The duration and voltage of tip pulses were edit in the software. For in‐plane PFM imaging, tips were modulated at 300 kHz with an AC voltage of 3 V. For the thermal testing combined with PFM, the sample was attached to a heating stage provided by Asylum Research. The heating and cooling speed was set to 5 °C min^−1^. After reaching the target temperature, temperature of the sample and heating stage was maintained for 15 min to obtain stabilization. In SS‐PFM testing, a Pt/Ir cantilever of spring constant 0.6 N m^−1^ (Rocky Mountian Nanotechnology 12PtIr400B) was used. An AC voltage of 5 V was applied to tips at 50 Hz. In SKPM testing, a Si cantilever with conducting Ti/Ir coating layer of spring constant 2 N m^−1^ (Asylum Research ASYELEC‐01) was used. An AC voltage of 5 V and DC bias of 8 V were applied to tips with distance between tips and sample surface set to 10 nm when collecting potential data.

## Conflict of Interest

The authors declare no conflict of interest.

## Supporting information

Supplementary
