# Supplementary material for: Flexible Robust and High‐Density FeRAM from Array of Organic Ferroelectric Nano‐Lamellae by Self‐Assembly
Source: Adv Sci (Weinh). 2019 Jan 28;6(6):1801931. doi: 10.1002/advs.201801931 (PMC6425439; doi:10.1002/advs.201801931)
Supplement: Supplementary file 1 — Supplementary [file ADVS-6-1801931-s001.pdf]

Copyright WILEY-VCH Verlag GmbH & Co. KGaA, 69469 Weinheim, Germany, 2019.

Supporting Information

for *Adv. Sci.,* DOI: 10.1002/advs.201801931

Flexible Robust and High-Density FeRAM from Array of Organic Ferroelectric Nano-Lamellae by Self-Assembly

*Mengfan Guo, Jianyong Jiang, Jianfeng Qian, Chen Liu, Jing Ma, Ce-Wen Nan, and Yang Shen**

Copyright WILEY-VCH Verlag GmbH & Co. KGaA, 69469 Weinheim, Germany,

2019.

Supporting Information

Flexible Robust and High-Density FeRAM from Array of Organic Ferroelectric Nano-lamellae by Self-Assembly

Mengfan Guo, Jianyong Jiang, Jianfeng Qian, Chen Liu, Jing Ma, Ce-Wen Nan and Yang Shen*

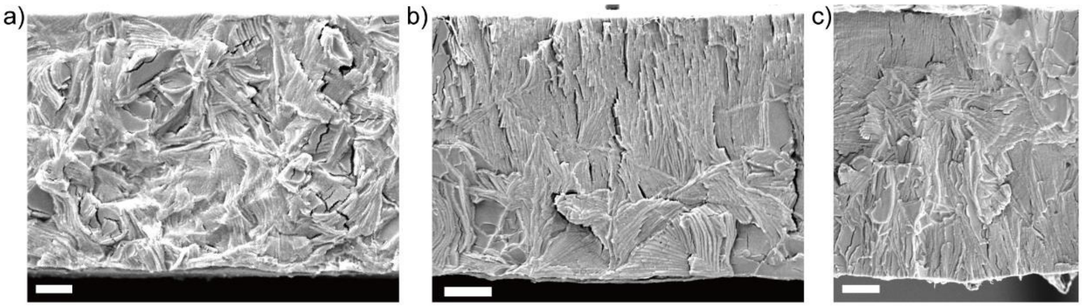

Figure S1. Cross sectional SEM images of thick SA and NSA films. a) NSA film. b) Film with self-assembly at one side. c) Film with self-assembly at both sides. Scale bars, 2 μm.

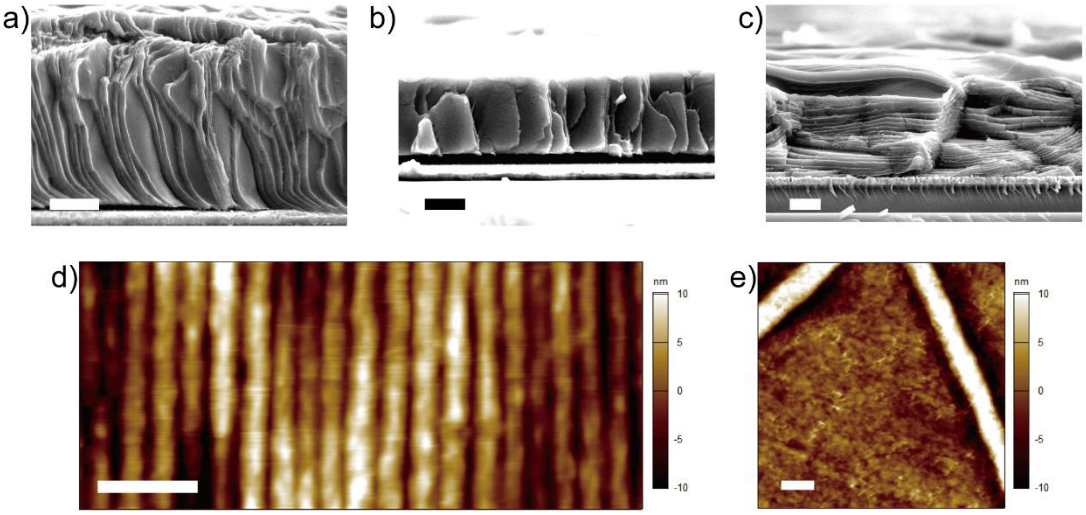

Figure S2. Cross sectional SEM images and surface AFM images of films on substrate. a,b) Cross sectional SEM images of a SA film with section nearly

perpendicular (a) and parallel (b) to lamellae. The scale bars are 500 nm. c) Cross sectional SEM images of a NSA film. As film thickness decreases to limited size, the lamellae tend to lie down generally. The scale bar is 500 nm. d,e) Surface AFM images of a SA film (d) and a NSA film (e). The scale bars are 400 nm.

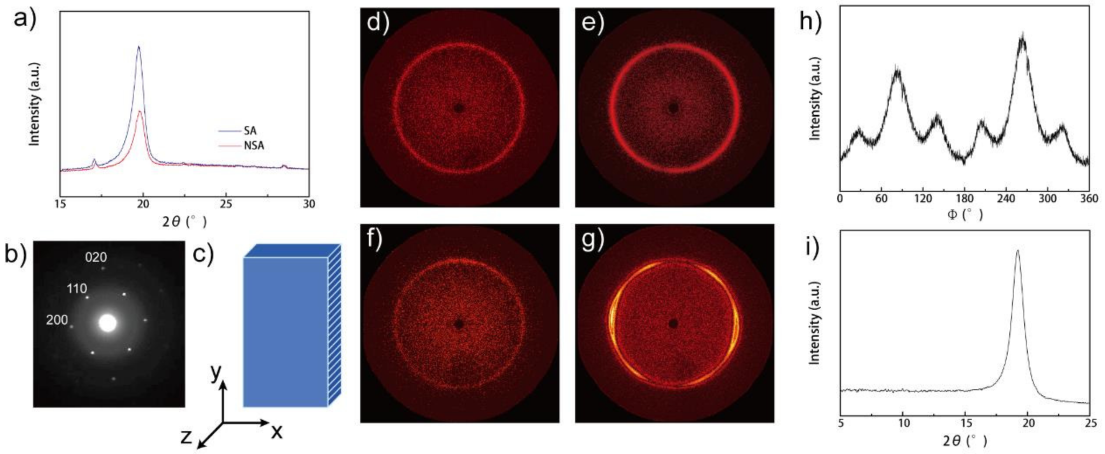

Figure S3. Diffraction identification of P(VDF-TrFE) films. a) XRD profiles of a SA film (blue) and a NSA film (red). b) SAED pattern of P(VDF-TrFE) films. c) Schematic illustration of X-ray direction and film position in WAXD experiment. d,e) 2D-WAXD identification of a NSA film with X-ray along Z (d) and X (e) direction. f,g) 2D-WAXD identification of a SA film with X-ray along Z (f) and X (g) direction. h,i) 1D-WAXD profiles of a SA film, obtained by integrating intensity along diffraction angle 2θ (h) and rotational angle Φ (i).

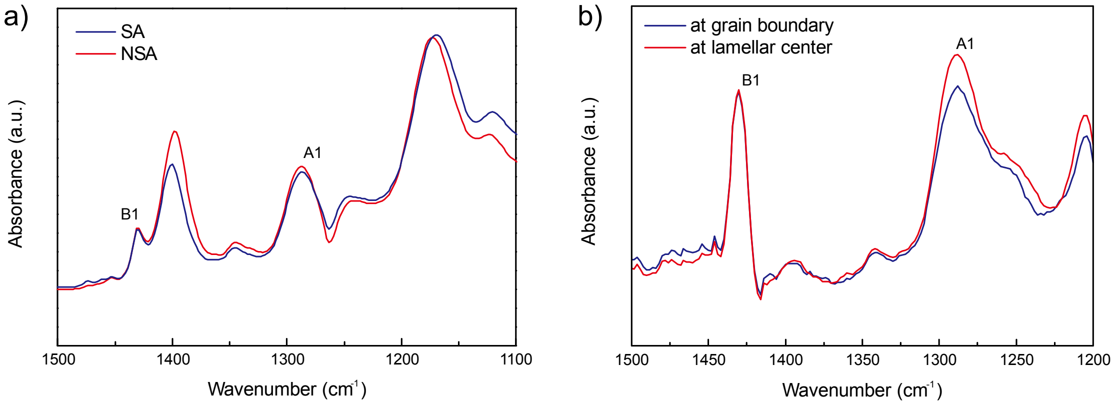

Figure S4. Infrared spectrum of P(VDF-TrFE) by Fourier transform method and AFM-IR method. a) FTIR spectrum of a SA film (blue) and a NSA film (red). b) Infrared spectrum of SA P(VDF-TrFE) obtained by AFM-IR. Two profiles correspond to area with stronger (blue) and weaker (red) absorbance at 1288 cm^{-1}.

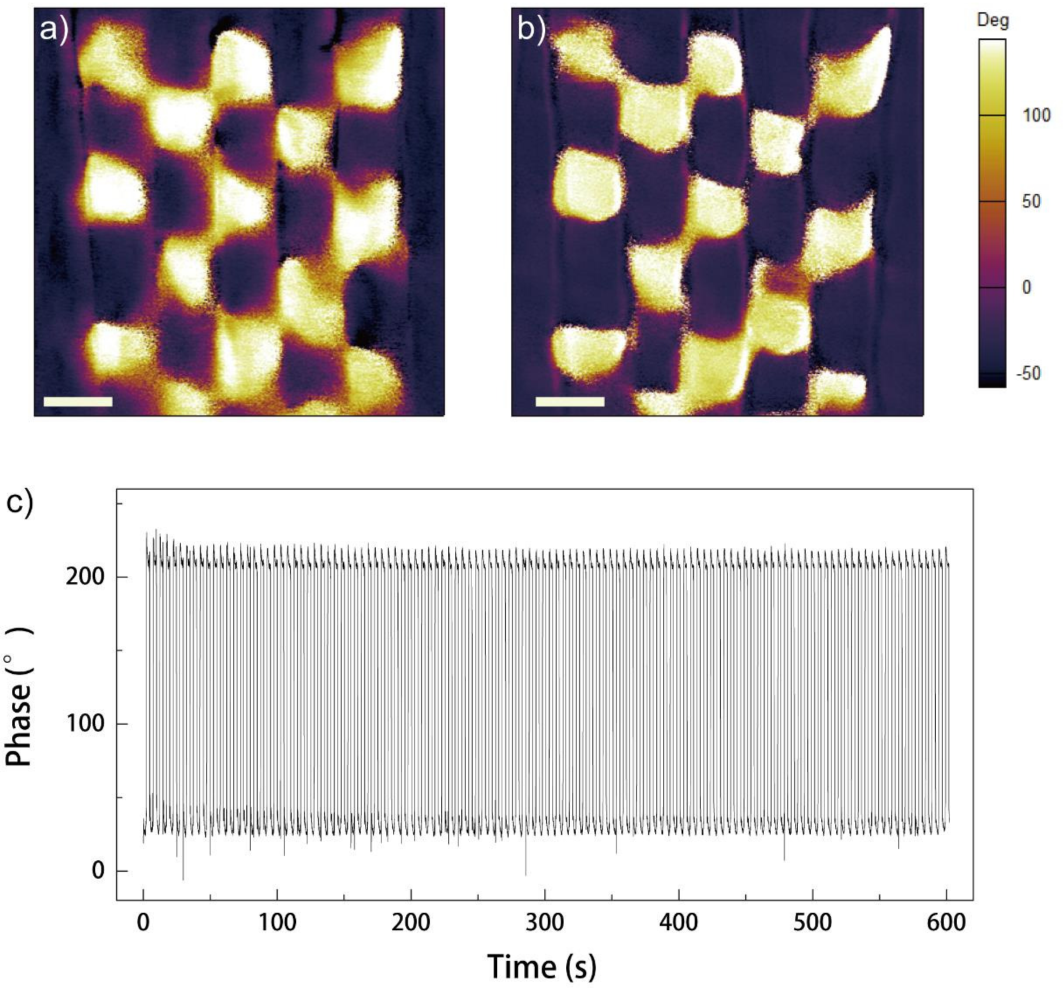

Figure S5. Retention and fatigue properties of SA P(VDF-TrFE) FeRAM. a,b) written data detected by PFM before (a) and after (b) a month. The scale bars, 100 nm. c) Fatigue behavior of a SA film after 100 switching of one point. The ferroelectric switching was generated and detected by SS-PFM.

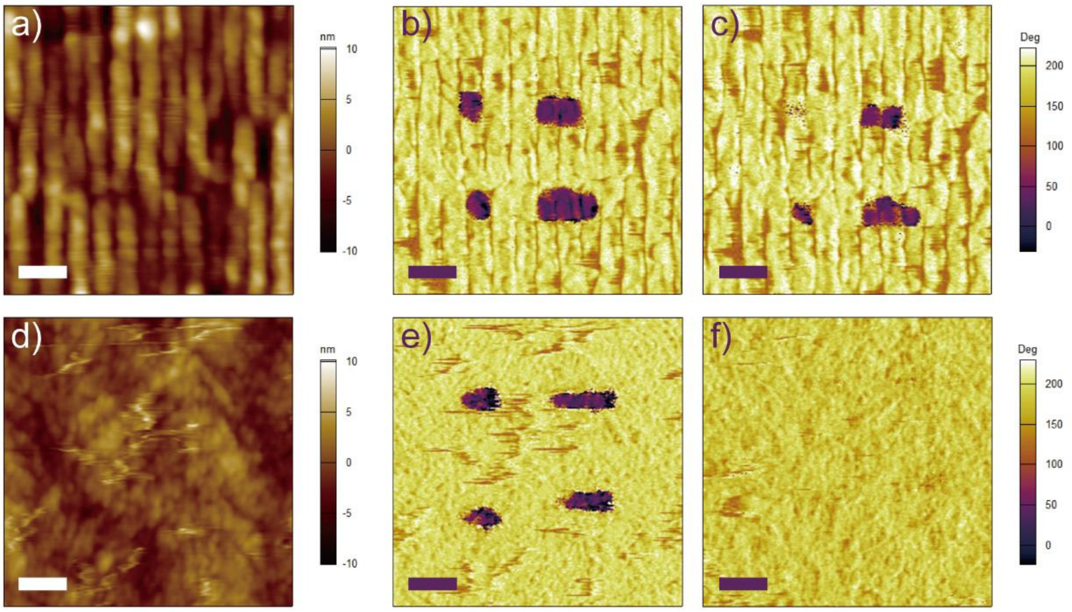

Figure S6. Thermal stability of P(VDF-TrFE) films. a-c) AFM topography image (a) of a SA film, and PFM phase image of reversal domains on it at 25 °C (b) and 90 °C (c). d-f) AFM topography image (d) of a NSA film, and PFM phase images of reversal domains on it at 25 °C (e) and 90 °C (f). The scale bars, 200 nm.

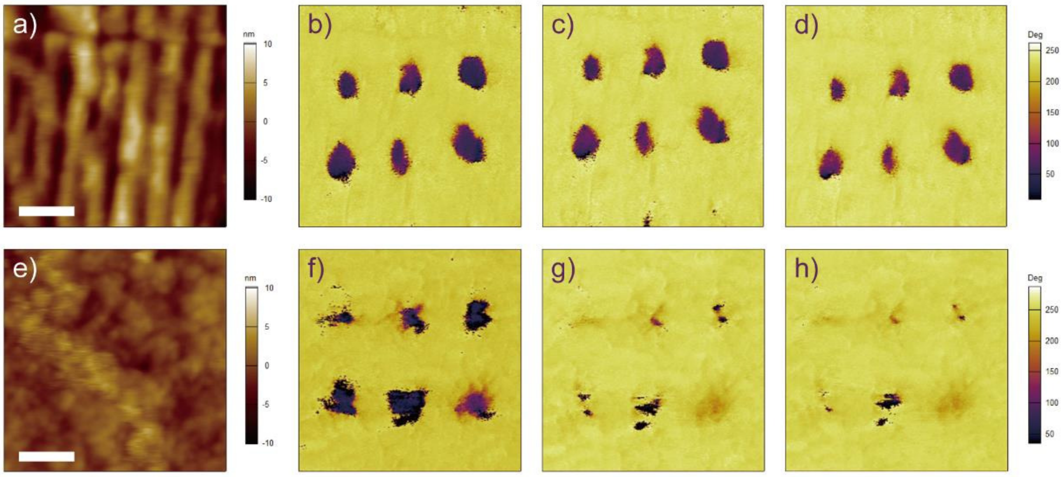

Figure S7. Thermal cycling ability of P(VDF-TrFE) films. a-d) AFM topography image (a) of a SA film, and PFM phase images of reversal domains on it before cycling (b), after the fourth (c) and twelfth (d) cycle. e-h) AFM topography image (e) of a NSA film, and PFM phase images of reversal domains on it before cycling (f), after the fourth (g) and twelfth (h) cycle. The scale bars, 200 nm.

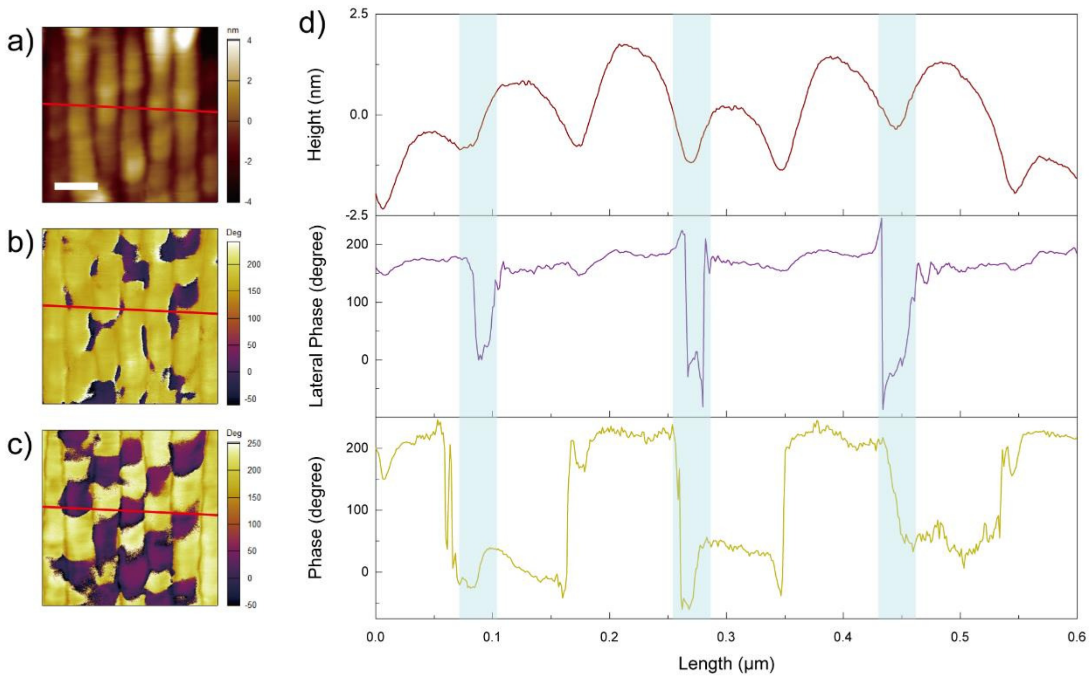

Figure S8. Topography and phase of SA P(VDF-TrFE). a) AFM topography, b) PFM in-plane phase, c) PFM out-of-plane phase images of SA P(VDF-TrFE) with reversal domains after retention of one month. d) The line section profiles corresponding to the red lines in (a-c). Stripe domains from in-plane signals are 180°, and overlap with anti-domain walls from out-of-plane signals at grain boundaries. The scale bar is 150 nm.

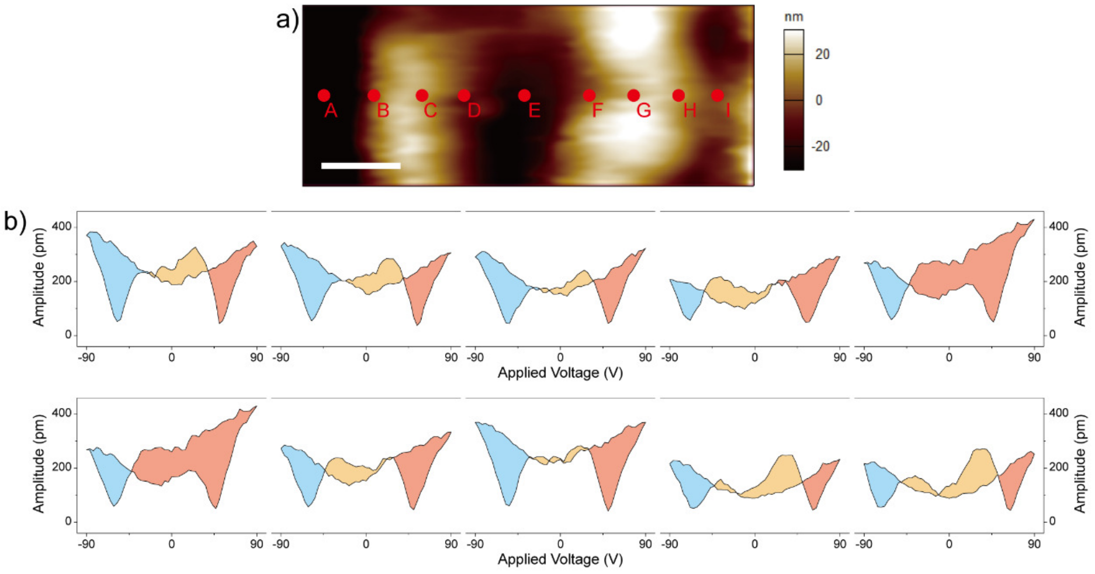

Figure S9. Switching spectra across two neighboring lamellae. a) AFM topography of a self-assembled with thickness around 2 μm. Switching experiments of a series of single points varying from the edge to the center of several lamellae, denoted by the

red spots, were conducted. Points A,E,I correspond to the crystal boundaries, and points C, G correspond to the center of lamellae. The scale bar is 150 nm. b,c) Switching spectra of points A-E (b) and E-F (c) crossing one edge-on lamella, respectively. Peaks of ferroelectric lamella are highlighted in red and blue, while additional peaks are highlighted in yellow. The spectra show the most asymmetry at crystal boundaries, and the most symmetry at center of lamellae, with generally two additional peaks at low voltage stimuli.

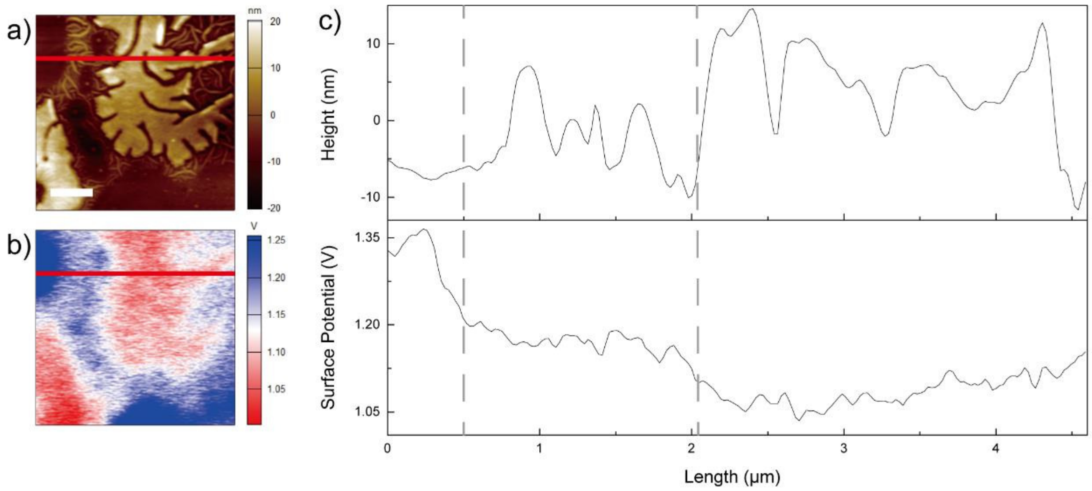

Figure S10. Surface Potential of a laid down lamella. a) AFM topography and b) SKPM images of a laid down lamella, with thickness around 20 nm. c) The line section profiles corresponding to the red lines in a and b. The dashed lines divide the profiles into three stages, with increasing height and decreasing surface potential. The scale bar is 100 nm.
